# Computed tomography findings of pneumonia resistant to empirical therapy in patients with hematologic diseases

**DOI:** 10.1590/0100-3984.2024.0118

**Published:** 2025-07-07

**Authors:** Luca Giuliani, Nicholas Landini, Giorgio Maria Masci, Silvia Palladino, Valeria Panebianco, Giammarco Raponi, Alice Di Rocco, Giuseppe Gentile, Carlo Catalano

**Affiliations:** 1 Policlinico Umberto I Hospital, Sapienza University, Rome, Italy.

**Keywords:** Pneumonia, Drug resistance, Hematologic neoplasms, Tomography, X-ray computed, Bronchoalveolar lavage, Pneumonia, Resistência a medicamentos, Neoplasias hematológicas, Tomografia computadorizada por raios X, Lavagem broncoalveolar

## Abstract

**Objective:**

To investigate computed tomography (CT) features of pneumonia that does not respond to empirical therapy in patients with hematologic diseases.

**Materials and Methods:**

This was a retrospective analysis of all patients with hematologic disease who were diagnosed with pneumonia between 2017 and 2023, did not respond to empirical therapy for the infection, and underwent bronchoalveolar lavage and CT within a week of each other. The distribution and CT pattern of pulmonary abnormalities were assessed, as was the presence of lymphadenopathy, pleural effusion, and pericardial effusion.

**Results:**

Forty-nine patients (30 males; mean age, 61 years) were included. We identified Gram-negative bacteria in 45 patients, Gram-positive bacteria in 13, and fungi in three. Pulmonary abnormalities were bilateral in 73% of the patients in the sample, and there was no difference in prevalence between the upper and lower lung fields in 53%. Common alterations were consolidation, in 73% of the patients, bronchial wall thickening, in 71%, bronchiectasis, in 55%, and nodules, in 53%; extrapulmonary findings were less common, being identified in ≤ 27%. Pulmonary findings were typically bilateral and without a predominance between the upper and lower lung fields (*p* < 0.05). Common associations were between consolidation and bronchiectasis, between nodules and bronchial wall thickening, and between bronchiectasis and bronchial wall thickening (*p* < 0.05 for all).

**Conclusion:**

The CT manifestations of pneumonia in patients with hematologic diseases not responding to empirical therapy can resemble those of lobular pneumonia with airway inflammation. For that reason, as well as because multiple pathogens can be present in the same patient, examination of bronchoalveolar lavage fluid can be necessary.

## Abstract (pt)

**Objetivo:**

Investigar características de tomografia computadorizada (TC) de pneumonia em pacientes com doenças hematológicas que não respondem à terapia empírica.

**Materiais e Métodos:**

Análise retrospectiva de todos os pacientes com doença hematológica diagnosticados com pneumonia entre 2017 e 2023, que não responderam à terapia empírica e foram submetidos a lavagem broncoalveolar e TC com intervalo de uma semana entre si. A distribuição e o padrão tomográfico das anormalidades pulmonares foram avaliados, assim como a presença de linfadenopatia, derrame pleural e derrame pericárdico.

**Resultados:**

Quarenta e nove pacientes (30 homens; idade média de 61 anos) foram incluídos. Foram identificadas bactérias Gram-negativas em 45 pacientes, bactérias Gram-positivas em 13 e fungos em três. As anormalidades pulmonares foram bilaterais em 73% dos pacientes da amostra, e não houve diferença na prevalência entre os campos pulmonares superiores e inferiores em 53%. Alterações comuns foram consolidação, em 73% dos pacientes, espessamento da parede brônquica, em 71%, bronquiectasias, em 55%, e nódulos, em 53%; achados extrapulmonares foram menos comuns, sendo identificados em ≤ 27%. Os achados pulmonares foram tipicamente bilaterais e sem predomínio entre os campos pulmonares superiores e inferiores (*p* < 0,05). As associações comuns foram entre consolidação e bronquiectasia, entre nódulos e espessamento da parede brônquica e entre bronquiectasia e espessamento da parede brônquica (*p* < 0,05 para todos).

**Conclusão:**

As manifestações tomográficas de pneumonia em pacientes com doenças hematológicas que não respondem à terapia empírica podem assemelhar-se às de pneumonia lobular com inflamação das vias aéreas. Por esse motivo, além da possibilidade de múltiplos patógenos estarem presentes no mesmo paciente, o exame do lavado broncoalveolar pode ser necessário.

## INTRODUCTION

Hematologic malignancies encompass a wide spectrum of blood cancers leading to disruptions in normal hematopoietic function. They comprise myeloid and lymphoid tumors, each with distinct subtypes. Common subtypes include leukemia, multiple myeloma, non-Hodgkin lymphoma, and Hodgkin lymphoma^(1)^. The number of incident cases of hematologic malignancies worldwide stood at 1,343,850 cases in 2019. In that same year, the age-standardized death rates for leukemia, multiple myeloma, non-Hodgkin lymphoma, and Hodgkin lymphoma were 4.26, 1.42, 3.19, and 0.34 per 100,000 population, respectively^(2)^.

Pulmonary complications are common causes of morbidity and mortality in patients with hematologic diseases^(3^,^4)^. There are several predisposing factors for the development of pulmonary infections in patients with hematologic diseases^(5)^, e.g., prolonged neutropenia^(6)^, intense immunosuppression, nosocomial exposures and postoperative complications, particularly in the lung transplant population^(7)^.

Pneumonia is typically caused by infection with Gram-positive bacteria, Gram-negative bacteria, viruses, or fungi^(6)^, with an overall mortality rate of 20–30%^(8^–^10)^. Staphylococcal infections account for 40–60% of infections with Gram-positive bacteria in patients with hematologic diseases. Among Gram-negative bacteria, *Escherichia coli* and *Pseudomonas aeruginosa* are the most common pathogens and can lead to severe conditions. In addition, infections with multidrug-resistant pathogens are particularly dangerous and can develop in patients who have received prolonged treatment with antibiotics^(11^–^13)^. The most common viral infections are those caused by herpesviruses, including cytomegalovirus and varicella-zoster virus^(14^,^15)^. Regarding fungal infections, although *Candida albicans* is prevalent, new strains of non-albicans *Candida* spp. with a more aggressive clinical character have been isolated. Among molds, *Aspergillus* spp. play a predominant role^(16)^.

Computed tomography (CT) of the chest is the imaging modality of choice for evaluating the lung parenchyma, involved in the diagnosis and monitoring of patients with lung disease^(17)^. The CT findings of pulmonary infections may vary, depending on the causative agent. Based on the radiological patterns, pneumonia can be classified as one of four main types^(18^,^19)^: lobar pneumonia; lobular pneumonia (bronchopneumonia); atypical pneumonia (interstitial pneumonia); and pneumonia with a predominantly nodular pattern.

Lobar pneumonia, such as that due to infection with *Streptococcus pneumoniae*, *Chlamydia pneumoniae*, *Mycoplasma pneumoniae*, or *Klebsiella pneumoniae*^(18)^, usually manifests as distinct, well-defined consolidation with a visible inner airway lumen, resulting in the characteristic air bronchogram sign^(19)^. The predominant pathogens causing lobular pneumonia are *Staphylococcus aureus* and *P. aeruginosa*. This type of pneumonia typically appears as patchy centrilobular or peribronchial nodules initially and can progress to dense consolidation over time^(19)^. Atypical pneumonia, or interstitial pneumonia, is characterized by small, focal or diffuse heterogeneous opacities evenly distributed throughout the affected lung. Those opacities are frequently described as ground-glass opacities with a reticular or reticulonodular pattern^(20)^.

Atypical types of bacterial pneumonia that affect immunocompetent patients account for approximately 15% of all cases of community-acquired pneumonia. The primary nonzoonotic pathogens responsible for these atypical bacterial infections include *M. pneumoniae*, *C. pneumoniae*, and *Legionella pneumophila*. Various zoonotic bacteria, notably *Chlamydia psittaci, Francisella tularensis*, and *Coxiella burnetii*, also play a significant role in the etiology of community-acquired pneumonia within certain immunocompetent populations. In addition, pneumonia resulting from infection with a viral pathogen is consistently regarded as a component of the atypical pneumonia category^(21)^. Among opportunistic infections, invasive pulmonary aspergillosis is a common complication in patients with neutropenia^(22)^. Distinctive signs of the angio-invasive forms of aspergillosis include a large pulmonary nodule surrounded by ground glass (known as the halo sign) and triangular pleural-based consolidations, usually representing hemorrhagic pulmonary infarction. Furthermore, the airway-invasive forms present centrilobular “tree-in-bud” nodules or confluent peribronchial thickening. Nodules with a halo sign constitute an early indication of angioinvasive aspergillosis and often grow despite appropriate therapy; when the inflammatory process becomes effective, areas of cavitation usually appear, leading to *restitutio ad integrum* or a scar.

Given the challenges in identifying the cause of pulmonary infiltrates in febrile patients with neutropenia, early, empirical administration of broad-spectrum antibacterial and, in some cases, antifungal therapy is crucial. This proactive approach enhances patient outcomes by addressing potential infections promptly^(23)^. Bronchoscopy with bronchoalveolar lavage (BAL) is a common procedure for the investigation of pulmonary infiltrates in such patients, especially when there is an unsatisfactory response to empirical treatment^(24^,^25)^, so that the specific therapy can be started as soon as possible.

Overall, BAL is thought to offer valuable diagnostic insights, and some studies have shown that the procedure has an acceptable safety profile in this specific patient population^(26^,^27)^. However, it is an invasive procedure, whereas CT is a largely noninvasive diagnostic tool, albeit one that involves exposure to ionizing radiation, which can be relevant in young patients requiring lifelong follow-up. Therefore, investigating the typical CT features of pneumonias resistant to empiric therapy may be relevant for clinical practice. In addition, the introduction of photon-counting CT could reduce the burden of radiation exposure^(28)^.

Considering the wide spectrum of infections that may affect individuals with hematologic disorders and their burden in patient management, we aimed to investigate the CT manifestations of pneumonia that is resistant to empirical therapy, related to the infective agents identified by BAL, looking for specific characteristics that may help radiologists in identifying the nature of the infection.

## MATERIALS AND METHODS

Patients were considered eligible for enrollment if they had been followed in the Hematology Department of Policlinico Umberto I Hospital, operated by Sapienza University, in Rome, Italy, between 2017 and 2023, had received a clinical diagnosis of pneumonia resistant to empirical therapy, and had consequently undergone BAL. The inclusion criteria were having undergone CT within a week of the BAL procedure, and the CT examination having involved the use of lung and soft tissue reconstruction kernels, with slice thicknesses ≤ 1.5 mm and ≤ 3mm, respectively. Patients with negative BAL results were excluded, as were those with negative CT results or CT findings not confidently attributable to an infection. Demographic and clinical data, including age, sex, hematologic disease, and drug therapy, as well as BAL results, were collected. Infection with *Candida* spp. was not investigated. This study was conducted in accordance with the principles of the Declaration of Helsinki and was approved by the Research Ethics Committee of Sapienza University (Reference no.7226; Protocol no. 0473/2024; dated May 23, 2024).

### CT analysis

Two radiologists (a chest radiologist with eight years of experience and a general radiologist with one year of experience) evaluated all CT scans by consensus in order to identify the CT features of pneumonia. They then assessed the location of abnormalities (as unilateral or bilateral), and their predominant pattern of distribution (as upper/lower, anterior/posterior, or central/peripheral). The carina was adopted as the anatomical landmark dividing the upper and lower lung fields as well as the anterior and posterior regions. The peripheral lung was defined as two or three rows of secondary pulmonary lobules, forming a layer of 3–4 cm at the lung periphery, whereas the central lung was defined as the sum of the remaining parts, as previously described^(29^–^32)^.

Patterns of parenchymal abnormalities—consolidation, ground glass, reticulation, and nodules with or without cavitation^(33)^—and airway alterations—bronchial wall thickening, mucoid impaction, mosaic pattern^(34)^—were evaluated as present or absent. The following extrapulmonary findings were also recorded^(34)^: lymphadenopathies (short axis > 1 cm); pleural effusion; and pericardial effusion. Images were analyzed with standard lung or soft tissue window settings, depending on the abnormality to be assessed. Coronal and sagittal multiplanar reconstructions were also available in all cases.

### Statistical analysis

Descriptive statistics were employed to summarize and present the data, using the absolute number and percentage or the mean and standard deviation. Chi-square tests were employed to assess the statistical significance of the differences observed. The statistical analysis was conducted by independently combining the distributions of infectious foci and the abnormalities, if present in ≥ 50% of patients, and was repeated for Gram-negative infections. Because there were few other infective agents, no additional sub-analyses were performed. For two-tailed tests, values of *p* < 0.05 were considered statistically significant^(35)^. This approach allows for a comprehensive evaluation of the data and enables the identification of meaningful associations.

## RESULTS

We initially recruited 60 patients in whom BAL and CT had been performed within a week of each other. A total of 11 patients were excluded, because of negative BAL results (n = 5) or negative CT results (n = 6). Therefore, the final sample comprised 49 patients. Of those 49 patients, 30 (61.2%) were men and 19 (38.8%) were women. The mean age was 61 years. The characteristics of the patients are shown in Table 1.

**Table 1:** Characteristics of and CT findings in patients with hematologic diseases and pneumonia that does not respond to empirical therapy (N = 49*).

Characteristic	All patients	Type of infection			
		Gram-negative bacteria	Gram-positive bacteria	Gram-negative and Grampositive bacteria	Gram-negative bacteria and fungal
Total, n (%)	49 (100)	33 (67.3)	4 (8.2)	9 (18.4)	3	(6.1)
Male, n (%)	30 (61.2)	22 (44.9)	2 (4.1)	5 (10.2)	1	(2.0)
Age (years), mean	61	64	59	63		67
Hematologic disease, n (%)						
• Non-Hodgkin lymphoma	20 (40.8)	16 (32.6)	1 (2.0)	1 (2.0)	2	(4.1)
• Acute myeloid leukemia	12 (24.5)	9 (18.4)	0 (0.0)	3 (6.1)	0	(0.0)
• Acute lymphoblastic leukemia	5 (10.2)	3 (6.1)	2 (4.1)	0 (0.0)	0	(0.0)
• Chronic lymphocytic leukemia	3 (6.1)	2 (4.1)	0 (0.0)	0 (0.0)	1	(2.0)
• Myelodysplastic syndrome(s)	3 (6.1)	1 (2.0)	0 (0.0)	2 (4.1)	0	(0.0)
• Primary myelofibrosis	2 (4.1)	1 (2.0)	0 (0.0)	1 (2.0)	0	(0.0)
• Hodgkin lymphoma	1 (2.0)	1 (2.0)	0 (0.0)	0 (0.0)	0	(0.0)
• Multiple myeloma	1 (2.0)	0 (0.0)	1 (2.0)	0 (0.0)	0	(0.0)
• Immune thrombocytopenia	1 (2.0)	0 (0.0)	0 (0.0)	1 (2.0)	0	(0.0)
• Bone marrow aplasia	1 (2.0)	0 (0.0)	0 (0.0)	1 (2.0)	0	(0.0)
CT findings					
• Distribution/location, n (%)					
- Unilateral	13 (26.5)	8 (16.3)	1 (2.0)	4 (8.2)	0	(0.0)
- Bilateral	36 (73.5)	25 (51.0)	3 (6.1)	5 (10.2)	3	(6.1)
- Anterior	8 (16.3)	5 (10.2)	0 (0.0)	3 (6.1)	0	(0.0)
- Posterior	19 (38.8)	10 (20.4)	3 (6.1)	4 (8.2)	2	(4.1)
- No predominance	22 (44.9)	18 (36.7)	1 (2.0)	2 (4.1)	1	(2.0)
- Central	18 (36.7)	11 (22.4)	2 (4.1)	4 (8.2)	1	(2.0)
- Peripheral	17 (34.7)	10 (20.4)	1 (2.0)	4 (8.2)	2	(4.1)
- No predominance	14 (28.6)	12 (24.5)	1 (2.0)	1 (2.0)	0	(0.0)
- Upper	4 (8.2)	3 (6.1)	0 (0.0)	1 (2.0)	0	(0.0)
- Lower	19 (38.8)	12 (24.5)	3 (6.1)	4 (8.2)	0	(0.0)
- No predominance	26 (53.1)	18 (36.7)	1 (2.0)	4 (2.0)	3	(6.1)
• Parenchyma, n (%)						
- Consolidation	36 (73.5)	25 (51.0)	2 (4.1)	6 (12.2)	3	(6.1)
- Ground-glass opacity	16 (32.6)	12 (24.5)	2 (4.1)	2 (4.1)	0	(0.0)
- Nodule(s)	26 (53.1)	17 (34.7)	3 (6.1)	4 (8.2)	2	(4.1)
- Cavitary	3 (6.1)	2 (4.1)	0 (0.0)	1 (2.0)	0	(0.0)
- Reticulation(s)	17 (34.7)	13 (26.5)	0 (0.0)	2 (4.1)	2	(4.1)
• Airways, n (%)						
- Bronchial wall thickening	35 (71.4)	24 (49.0)	4 (8.2)	4 (8.2)	4	(8.2)
- Bronchiectasis	27 (55.1)	19 (38.8)	3 (6.1)	2 (4.1)	3	(6.1)
- Mosaic attenuation/air trapping	9 (18.4)	7 (14.3)	0 (0.0)	2 (4.1)	0	(0.0)
- Mucus plug(s)	19 (38.8)	11 (22.4)	3 (6.1)	2 (4.1)	3	(6.1)
• Other findings, n (%)						
- Lymph node enlargement	9 (18.4)	6 (12.2)	1 (2.0)	1 (2.0)	1	(2.0)
- Pericardial effusion	3 (6.1)	1 (2.0)	0 (0.0)	1 (2.0)	1	(2.0)
- Pleural effusion	13 (26.5)	9 (18.4)	1 (2.0)	3 (6.1)	0	(0.0)

* All percentages shown in the table are based on the total number of patients in the sample, rather than on the total number in each subgroup/column.

On BAL, 25 patients (51.0%) tested positive for only one pathogen, 18 (36.7%) tested positive for two, and six (12.2%) tested positive for three or more. Among those who tested positive for only one pathogen, the most common was *P. aeruginosa*, which was identified in nine patients (18.4%). The infection was sustained by one or more species of Gram-negative bacteria in 33 patients (67.3%), by Gram-negative and Gram-positive bacteria in 9 (18.4%), and only by Gram-positive bacteria in four (8.2%). Fungal infection was detected in three patients (6.1%) and was accompanied by Gram-negative bacterial infection in all three. The BAL results are shown in Table 2.

**Table 2:** BAL results.

Number of patients		Pathogen(s) identified		
9	*Pseudomonas aeruginosa*	-	--	--
3	*Stenotrophomonas maltophilia*	-	-	-
2	*Staphylococcus haemolyticus*	-	-	-
1	*Enterococcus faecium*	-	-	-
1	*Enterobacter aerogenes*	-	-	-
1	*Haemophilus parainfluenzae*	-	-	-
1	*Pseudomonas putida*	-	-	-
1	*Escherichia coli*	-	-	-
1	*Non-fermenting gram-negative*	-	-	-
1	*Haemophilus influenzae*	-	-	-
1	*Klebsiella pneumoniae*	-	-	-
1	*Enterococcus faecalis*	-	-	-
1	*Enterobacter cloacae/asburiae*	-	-	-
1	*Haemophilus haemolyticus*	-	-	-
2	*Klebsiella pneumoniae*	*Pseudomonas aeruginosa*	-	-
2	*Klebsiella pneumoniae*	*Stenotrophomonas maltophilia*	-	-
1	*Enterococcus faecalis*	*Pseudomonas aeruginosa*	-	-
1	*Stenotrophomonas maltophilia*	*Geotrichum sp.*	-	-
1	*Stenotrophomonas maltophilia*	*Enterococcus faecalis*	-	-
1	*Stenotrophomonas maltophilia*	*Pseudomonas aeruginosa*	-	-
1	*Stenotrophomonas maltophilia*	*Pseudomonas aeruginosa*	-	-
1	*Aspergillus fumigatus*	*Klebsiella pneumoniae*	-	-
1	*Acinetobacter junii*	*Pseudomonas mendocina*	-	-
1	*Streptococcus agalactiae*	*Pseudomonas aeruginosa*	-	-
1	*Enterococcus faecium*	*Klebsiella pneumoniae*	-	-
1	*Aspergillus fumigatus*	*Pseudomonas aeruginosa*	-	-
1	*Acinetobacter baumannii*	*Pseudomonas aeruginosa*	-	-
1	*Enterococcus faecium*	*Pseudomonas aeruginosa*	-	-
1	*Enterococcus faecium*	*Pseudomonas mendocina*	-	-
1	*Stenotrophomonas maltophilia*	*Acinetobacter baumannii*	-	-
1	*Stenotrophomonas maltophilia*	*Nocardia sp.*	*Staphylococcus haemolyticus*	-
1	*Stenotrophomonas maltophilia*	*Pseudomonas aeruginosa*	*Klebsiella pneumoniae*	-
1	*Stenotrophomonas maltophilia*	*Pseudomonas aeruginosa*	*Enterobacter aerogenes*	-
1	*Stenotrophomonas maltophilia*	*Pseudomonas aeruginosa*	*Enterococcus faecalis*	-
1	*Stenotrophomonas maltophilia*	*Pseudomonas aeruginosa*	*Haemophilus parainfluenzae*	-
1	*Stenotrophomonas maltophilia*	*Pseudomonas sp.*	*Staphylococcus haemolyticus*	*Klebsiella pneumoniae*

### CT analysis

**Figure 1:**
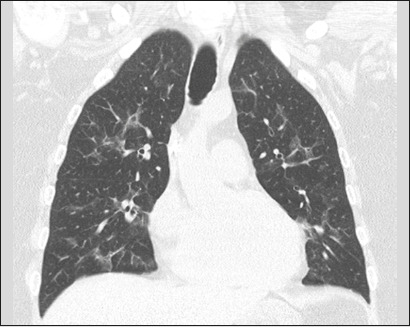
A 54-year-old male with pneumonia caused by infection with *S. maltophilia* and *E. faecalis*. CT showing bilateral ground-glass opacities without a predominance between the upper and lower lung fields.

**Figure 2:**
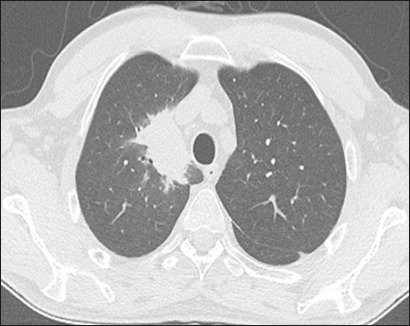
A 65-year-old male with pneumonia caused by infection with *E. aerogenes*. CT showing consolidation in the upper right lung field.

**Figure 3:**
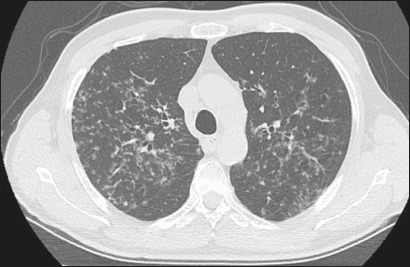
A 60-year-old female with pneumonia caused by infection with *E. faecalis*. CT showing bilateral nodules.

**Figure 4:**
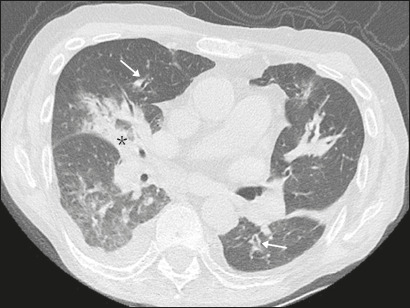
A 47-year-old female with pneumonia caused by infection with *P. aeruginosa*, *K. pneumoniae*, and *S. maltophilia*. CT showing bilateral consolidations (asterisk) and bronchial wall thickening (arrows). Pleural effusions are also present.

As illustrated in Figure 1, the CT features of pneumonia were bilateral in 36 patients (73.5%) and there was no predominance between the upper and lower lung fields in 26 (53.1%). Figure 2 depicts consolidation, which was seen in 36 (73%) patients (73.5%), Figure 3 shows nodules, which were seen in 26 (53.1%), and Figure 4 illustrates bronchial wall thickening, which was seen in 35 (71.4%). As shown in Table 1, we observed bronchiectasis in 27 patients (55.1%), pleural effusion in 13 (26.5%), lymphadenopathies in nine (18.4%), and pericardial effusion in three (6.1%).

Of the 49 patients in our sample, 33 (67.3%) were infected with Gram-negative bacteria. Abnormalities showed no predominance between the upper or lower lung fields or between unilateral and bilateral (*p* = 0.002), especially when only infection with Gram-negative pathogens was considered (*p* = 0.006). The most common combination was consolidation with bronchiectasis (*p* = 0.006), even when only infection with Gram-negative pathogens was considered (*p* = 0.03), followed by nodules with bronchial wall thickening (*p* = 0.005; Gram-negative, *p* = 0.03) and bronchial wall thickening with bronchiectasis (*p* = 0.00002; Gram-negative, *p* = 0.009).

## DISCUSSION

In this study, we have identified the CT features of pulmonary infections that did not respond to empirical therapy in patients with hematologic diseases who underwent BAL to identify the pathogen.

The BAL technique is minimally invasive and offers valuable insights into the pulmonary microbiome, being particularly beneficial in the diagnostic workup of patients with hematologic malignancies who exhibit respiratory complications. When empirical antibiotic therapy fails to yield clinical improvement, BAL allows for direct sampling of the lower respiratory tract, facilitating the identification of specific infectious agents responsible for respiratory symptoms. Early intervention may be pivotal in guiding targeted therapy, particularly in scenarios in which rapid identification of the pathogen is necessary for effective management.

Overall, BAL is an integral component of the diagnostic algorithm for patients with hematologic disorders facing respiratory challenges, particularly when initial empirical treatments prove ineffective. Its role in achieving a definitive diagnosis can significantly alter the therapeutic trajectory, improving clinical outcomes in this vulnerable population.

In our cohort of patients with treatment-resistant pneumonia who underwent BAL, most of the infections were with Gram-negative bacteria, the most common being *P. aeruginosa*. However, cases in which *Candida* sp. was identified in the BAL fluid were not considered, given the rarity of *Candida* pneumonia and the lack of methods that can differentiate between commensalism, colonization, and infection with *Candida* sp.^(36)^.

In our study sample, the abnormalities seen on CT were commonly bilateral and there was no predominance in distribution between the upper and lower lung fields. In addition, consolidation, nodules, bronchial wall thickening, and bronchiectasis were common findings.

As for the parenchymal distribution of the CT findings, abnormalities without a predominance between the upper and lower lung fields were commonly seen in combination with a bilateral distribution, as were consolidations in combination with bronchiectasis, nodules in combination with bronchial wall thickening, and bronchial wall thickening in combination with bronchiectasis. These characteristics may resemble those of lobular pneumonia with signs of airway inflammation. These results may match the BAL results but can be considered nonspecific for any diagnosis. However, to determine whether these findings would be helpful in clinical practice, a comparative analysis of the CT patterns exhibited by patients who respond positively to empirical therapy is desirable. If the CT findings in this clinical group differ from those observed in patients with treatment-resistant pneumonia, it would be significant for clinical practice.

Nouri et al.^(37)^ conducted a cross-sectional study of patients with hematologic malignancies and acute lung symptoms. The most common radiological findings included pleural effusion (in 42.0%), mediastinal lymphadenopathy (in 38.5%), consolidation (in 37.0%), ground-glass opacities (in 33.5%), and nodules (in 22.0%). The authors found that the distribution of consolidation was more often segmental and unilateral, whereas ground-glass opacities were also more commonly segmental but bilateral. In another study, Burivong et al.^(38)^ examined patients with hematologic malignancies who experienced episodes of febrile neutropenia. They identified the following common CT patterns: pulmonary consolidation (in 56.0%), ground-glass attenuation (in 40.0%), and nodules or masses (in 32.0%). In contrast with the findings of previous studies, we found that, in patients with hematologic diseases, consolidation and nodules were the most common parenchymal abnormalities, followed by ground-glass opacities. However, the high frequency of bronchiectasis in our study sample is likely linked to previous infections in predisposed patients. Recurrent infection can lead to chronic, irreversible changes in the airways. A significant percentage of patients exhibit bronchial wall thickening, which is an inflammatory sign in the airways and represents a major alteration, as does consolidation in the lung parenchyma.

The reported prevalence of polymicrobial pulmonary infection in patients with hematologic malignancies and pulmonary infiltrates detected through BAL ranges from 20% to 60%^(39^–^42)^. In our study, the prevalence of polymicrobial infection was 49%, which aligns with the findings of other researchers.

In various studies of patients with hematologic diseases undergoing BAL for pathogen identification, the primary pathogen identified was *Aspergillus* sp. Other common pathogens include *S. pneumoniae* and *Pneumocystis jirovecii*^(43^–^49)^. However, in our study, the majority of infections (67%) were caused by Gram-negative bacteria, with *P. aeruginosa* being the most frequently identified.

One of the risk factors for *Pseudomonas* infections is neutropenia, a typical, prolonged condition in patients with hematologic diseases. In our clinical practice, the first-line agent for the empirical treatment of febrile neutropenia is cefepime, with piperacillin-tazobactam and meropenem being second- and third-line agents, respectively.

Bergas et al.^(50)^ conducted a matched-cohort study about the use of ceftolozane-tazobactam for the treatment of bloodstream infection with *P. aeruginosa* in patients with hematologic diseases who develop neutropenia. The authors found that patients treated with ceftolozane-tazobactam were less likely to need mechanical ventilation (13.6% vs. 33.3%), as well as that 7-day and 30-day mortality were lower in the ceftolozane-tazobactam-treated group (6.8% vs. 34.1% and 22.7% vs. 48.9%, respectively). Therefore, if studies involving larger cohorts confirm *Pseudomonas* as a major agent of pneumonia resistant to empirical therapy, ceftolozane-tazobactam could be considered as a second-line treatment in clinical practice.

The differences between our results and those of other authors are likely due to the distinct design of our study, in which BAL was performed only in patients who did not respond to empirical therapy. In addition, at our center, we adopted a specific therapy for *Aspergillus* infection when CT scans and laboratory tests confirmed a diagnosis of invasive aspergillosis. In fact, invasive aspergillosis may be suspected in patients with neutropenia when there are distinct CT characteristics and laboratory findings, such as the presence of the *Aspergillus* galactomannan antigen and 1,3-beta-D-glucan in the blood. The low number of patients infected with *Aspergillus* in our sample may have also affected the results, particularly regarding the specific signs of invasive aspergillosis. However, we adhered to the clinical practices of our center to ensure that our findings would be reliable for daily routine.

Infection with the most common pathogen in our sample, *P. aeruginosa*, usually manifests as consolidations in the upper lung fields and nodules, in 80% and 50% of cases, respectively^(51)^. In our study, CT abnormalities in patients infected with *P. aeruginosa* were bilateral in the majority of cases and without a difference in distribution between the upper and lower lung fields in more than half. We observed consolidation in 78% of the patients and nodules in 55% (data for individual pathogens not shown). However, the small sample size precluded a sub-analysis or confident comparison with the literature.

The main limitations of our study are the retrospective design and the relatively small sample size. Although our study offers valuable preliminary insights, it calls for a cautious interpretation of the findings. Comprehensive future research involving larger and more diverse populations, comparing resistant and non-resistant cases of pneumonia, is warranted in order to validate our results and provide a more definitive understanding of the clinical implications.

In conclusion, in patients with hematologic diseases, pneumonia requiring BAL to identify the pathogen, especially after unsuccessful empirical therapy, may present as multifocal pneumonia (like lobular pneumonia) with signs of acute and chronic airway involvement. These infections are often caused by Gram-negative bacteria. Therefore, performing a BAL examination may still be essential due to the frequent presence of multiple, coexisting pathogens.
